# Animal model of naturally occurring bladder cancer: Characterization of four new canine transitional cell carcinoma cell lines

**DOI:** 10.1186/1471-2407-14-465

**Published:** 2014-06-25

**Authors:** Kusum Rathore, Maria Cekanova

**Affiliations:** 1 Department of Small Animal Clinical Sciences, The University of Tennessee, College of Veterinary Medicine, 2407 River Drive A122, Knoxville, TN 37996-4550, USA

**Keywords:** Transitional cell carcinoma, Canine, Xenograft, Bladder cancer

## Abstract

**Background:**

Development and further characterization of animal models for human cancers is important for the improvement of cancer detection and therapy. Canine bladder cancer closely resembles human bladder cancer in many aspects. In this study, we isolated and characterized four primary transitional cell carcinoma (K9TCC) cell lines to be used for future *in vitro* validation of novel therapeutic agents for bladder cancer.

**Methods:**

Four K9TCC cell lines were established from naturally-occurring canine bladder cancers obtained from four dogs. Cell proliferation rates of K9TCC cells *in vitro* were characterized by doubling time. The expression profile of cell-cycle proteins, cytokeratin, E-cadherin, COX-2, PDGFR, VEGFR, and EGFR were evaluated by immunocytochemistry (ICC) and Western blotting (WB) analysis and compared with established human bladder TCC cell lines, T24 and UMUC-3. All tested K9TCC cell lines were assessed for tumorigenic behavior using athymic mice *in vivo*.

**Results:**

Four established K9TCC cell lines: K9TCC#1Lillie, K9TCC#2Dakota, K9TCC#4Molly, and K9TCC#5Lilly were confirmed to have an epithelial-cell origin by morphology analysis, cytokeratin, and E-cadherin expressions. The tested K9TCC cells expressed UPIa (a specific marker of the urothelial cells), COX-2, PDGFR, and EGFR; however they lacked the expression of VEGFR. All tested K9TCC cell lines confirmed a tumorigenic behavior in athymic mice with 100% tumor incidence.

**Conclusions:**

The established K9TCC cell lines (K9TCC#1Lillie, K9TCC#2Dakota, K9TCC#4Molly, and K9TCC#5Lilly) can be further utilized to assist in development of new target-specific imaging and therapeutic agents for canine and human bladder cancer.

## Background

Bladder cancer is the fourth most common cancer in men and the eighth most common malignancy in women in the US according to the ACS. An estimated 74,690 new cases of bladder cancers are expected to occur in 2014 in the US. An estimated 15,580 bladder cancer-related deaths will occur in 2014 in the US [1]. The early stage of bladder cancers is usually surgically removed followed by immuno- or chemotherapy [2]. However more advanced carcinomas may often require cystectomy [1]. Precise early detection of tumors and accurate monitoring of tumor response to treatment are keys for survival of patients [3]. Up to 70% of patients with non-muscle-invasive bladder cancer will develop a local recurrence after transurethral resection of the bladder tumor [2,4].

Canine transitional cell carcinomas (K9TCC) closely resemble human invasive urinary bladder cancers [5]. The urinary bladder cancer is an uncommon type of cancer in dogs, comprising < 2% of all reported canine malignancies [6]; however 97% of bladder tumors are malignant at the time of diagnosis. The bladder K9TCC is the most common neoplasm affecting the urinary tract of dogs [5]. The histologic and biologic characteristics of bladder cancers in dogs are similar to bladder cancers in humans [7,8]. Canine TCC are low grade with superficial papillary appearance or high grade invasive tumors that spreads through the bladder wall to lymph nodes and to other organs, such as liver and lung predominantly [5,7,9]. The exact cause of TCC in dogs is still not know, however a genetic predisposition, pesticides, insecticides, and second hand smoke are considered major factors [5,7,9]. Dogs diagnosed with spontaneous tumors offer a unique model of bladder cancer to study its development and evaluation of new therapies [10-12]. The chemically- or genetically-induced TCC tumors in rodent, do not completely represent human cancer. Very few primary K9TCC cell lines are currently available [11]; therefore, there is a further need for new primary K9TCC cell lines to better understand TCC. Primary TCC cell lines closer mimic the biological behavior of primary tumors as compared to established immortalized cell lines or cell lines kept for long time in culture. Cells in long term culture may accumulate gene mutations.

There are numerous studies that show the correlation of the expression profiles of tumor markers in K9TCC with human TCC. The uroplakins are species-conserved integral membrane proteins that are present on the apical membrane of the terminally-differentiated superficial urothelial cells of normal bladder and preserving their expressions in neoplastic bladder TCC [13-15]. On the other hand, COX-2 is overexpressed in human bladder cancers, but not present in normal urothelium [7]. COX-2 expression increases with the stage and grade of bladder cancer [16]. Various growth factor receptors are also used as markers for bladder cancer, e.g. platelet-derived growth factor receptor (PDGFR) is associated with progression of human bladder cancer [17]. The epidermal growth factor receptor (EGFR) is also overexpressed by many carcinomas, including bladder cancers [18]. Vascular endothelial growth factor receptor (VEGFR) is expressed not only in endothelial cells, but also in carcinoma cells [19,20].

The purpose of this study was to characterize primary K9TCC cell lines to better understand the mechanisms of canine and human bladder cancers. Here, we reported the characterization of four new primary K9TCC cell lines using Western blotting (WB) and immunocytochemistry (ICC) analysis *in vitro*. In addition, we confirmed tumorigenic behavior of all tested K9TCC cell lines using athymic mice model *in vivo*. New K9TCC cell lines might be used to further evaluate novel imaging and therapeutic agents for human and canine bladder cancers.

## Methods

### Antibodies and other reagents

Antibody for COX-2 was obtained from Cayman Chemical Corporation (Ann Arbor, MI); antibodies for vimentin, PDGFR, VEGFR, E-cadherin, cyclin D1, p27, p-ERK1/2, UPIa, actin, and secondary anti-goat were purchased from Santa Cruz Biotechnology (Santa Cruz, CA); antibodies for cytokeratin and Ki67 were obtained from Dako (Carpinteria, CA); antibody for p65 (NF-κB) was purchased from BD Biosciences (San Jose, California); and antibody for EGFR, secondary anti-rabbit, and anti-mouse antibodies were obtained from Cell Signaling (Boston, MA). All other chemicals and reagents were purchased from Thermo Fisher Scientific (Pittsburgh, PA), unless otherwise specified.

### Human cell lines

Human transitional cell carcinoma cell lines T24 and UMUC-3 were purchased from American Type Culture Collection (ATCC, Manassas, VA). Human T-24 cells were maintained in DMEM:Ham’s F12 mixture (1:1) and human UMUC-3 cells were maintained in EMEM media; respectively, supplemented with 10% fetal bovine serum, 100 I.U. penicillin, and 100 μg/mL streptomycin. Cells were grown in an atmosphere of 5% CO2 at 37°C.

### Canine transitional cell carcinomas (K9TCC)

Primary K9TCC cells were isolated from biopsy specimens obtained by cystoscopy from client-owned dogs diagnosed with bladder TCC. The cystoscopy was performed as a part of diagnosis with best practice of veterinary care through the Center for Minimally Invasive Procedures at the College of Veterinary Medicine of the University of Tennessee. Establishment of primary K9TCC cell lines procedure was in accordance with approved protocol by the University of Tennessee IACUC committee with client consent to use non-utilized tissue specimen for our research. The canine patients with bladder cancers had at time of diagnosis advance stages of TCC. The non-utilized biopsy tissues were washed, trypsinized (0.25% trypsin-EDTA for 2–5 min), and cultured in RPMI-1640 media with L-glutamine supplemented with 10% fetal bovine serum, 100 I.U. penicillin, and 100 μg/ml streptomycin in an atmosphere of 5% CO2 at 37°C for 24 hours. Colonies of epithelial cells identified under microscope were transferred into new culture dishes and expanded. K9TCC cells that progressed through 6 to 9 passages were characterized. K9TCC cells were cryo-preserved and recovered for tissue culture to confirm their viability. All cell lines have been maintained in the laboratory for longer than 15 passages.

### Doubling time of K9TCC cells

K9TCC cells were plated in triplicate in 6-well plates. Cells were trypsinized and counted using a hemocytometer 24, 48, and 72 hours after plating. The doubling time for the K9TCC cells was calculated using the formula dt = t X [ln2/ln(Ct/Co), where dt = doubling time, t = time between cell counts Ct and Co, Co = initial count, Ct = count after time t, and ln = natural log. Time (t) was expressed in hours.

### Cell morphology of K9TCC cells

K9TCC cells were grown in RPMI-1640 media and allowed to reach 60-70% confluence. The morphology was examined under phase-contrast microscope with 20 × objective magnification (Vistavision, VWR) and images were captured using Moticam camera (VWR) with Motic 5.0 software.

### Immunocytochemistry (ICC)

K9TCC cells were plated on 4-chamber slides (Lab-Tek II, Nalge Nunc, Naperville, IL) and cultured until they reached 80% to 90% confluence within 24–48 hours and followed the ICC protocol previously described [21]. Cells were fixed with 2% paraformaldehyde for 10 min at r.t. and followed by blocking using protein block solution for 30 min. K9TCC cells were incubated with primary antibodies (UPIa, vimentin, cytokeratin, COX-2, PDGFR, VEGFR, EGFR, and Ki67). The details about dilutions, exposure times, and temperatures are listed in the Table 1. Specific secondary antibodies using streptavidin-biotin detection system (BioGenex Laboratories, Inc., Fremont CA) were incubated for 30 min each, followed by visualization with a DAB substrate. Nuclei of cells were counter-stained by hematoxylin, slides were mounted, cover-slipped, and evaluated under Leitz DMRB microscope (Leica). The images were captured by DP73 camera (Hunt Optics and Imaging, Pittsburgh, PA) attached to microscope using cellSens software (Olympus). The percentage of the positive cells were calculated in three fields with 20 × magnification. The scoring of staining was done as following: +++ ≥75%; ++ = 75–50%; + = 50-25%; - ≤25% positive cells per field of view.

**Table 1:** List of primary antibodies used for IHC, ICC, and WB analysis

**Primary antibody**	**Antigen retrieval for IHC**	**Dilution & incubation time**			**Product number**	**Vendor**
		**IHC**	**ICC**	**WB**		
Anti-UPIa	none	1:100 o.n., 4°C	1:50 1 hour, r.t.	1:1000 o.n., 4°C	sc-15173 (C-18)	Santa Cruz Biotechnology, Santa Cruz, CA
Anti-Cytokeratin	Proteinase- K	1:800 1 hour, r.t.	1:50 1 hour, r.t.	1:1000 o.n., 4°C	M3515 (AE1/AE3)	Dako, Carpinteria, CA
Anti-EGFR	Sodium Citrate	1:100 o.n., 4°C	1:25 1 hour, r.t.	1:1000 o.n., 4°C	sc-03 (1005)	Cell Signaling Technology, Boston, MA
Anti-COX-2	Sodium Citrate	1:500 o.n., 4°C	1:50 1 hour, r.t.	1:1000 o.n., 4°C	160126	Cayman Chemical, Ann Arbor, MI
Anti-p65	none	1:500 1 hour, r.t.	-	1:1000 o.n., 4°C	610868	BD Transduction Laboratories, San Jose, CA
Anti-Vimentin	-	-	1:100 1 hour, r.t.	-	sc-7557 (C-20)	Santa Cruz Biotechnology, Santa Cruz, CA
Anti-Ki67	-	-	1:50 1 hour, r.t.	-	M7240 (MIB-1)	Dako, Carpinteria, CA
Anti-PDGFR	-	-	1:25 1 hour, r.t.	1:1000 o.n., 4°C	SC-338 (C-20)	Santa Cruz Biotechnology, Santa Cruz, CA
Anti-VEGFR	-	-	1:25 1 hour, r.t.	1:500 o.n., 4°C	2467 (55B11)	Cell Signaling Technology, Boston, MA
Anti-phospho-ERK1/2	-	-	-	1:1000 o.n., 4°C	sc-7383 (E-4)	Santa Cruz Biotechnology, Santa Cruz, CA
Anti-p27	-	-	-	1:1000 o.n., 4°C	sc-528 (C-19)	Santa Cruz Biotechnology, Santa Cruz, CA

Notes: o.n. - over night; r.t. - room temperature.

### Immunohistochemistry

Dissected tissues from athymic mice and primary tumor samples from dogs diagnosed with TCC were formalin-fixed and paraffin-embedded and sectioned at 7 μm. Hematoxylin and eosin (H&E), and IHC staining was performed following standard protocols [21]. After deparaffinization, the antigen retrieval step was performed as listed in Table 1, followed by blocking of non-specific binding. Tissues were incubated with primary antibodies (UPIa, COX-2, cytokeratin, EGFR, and p65) according the conditions listed in Table 1, followed by the incubation with the specific secondary antibodies using streptavidin/biotin detection system and visualized by DAB staining. Nuclei were counter-stained with hematoxylin, slides were cover-slipped, and evaluated using Leitz DMRB microscope. The images were captured by DP73 camera attached to microscope using cellSens software (Olympus).

### Western blotting (WB)

Human and K9TCC cells were cultured in media with or without serum for 24 hours. After incubation, the cells were lysed in ice-cold RIPA buffer supplemented with protease and phosphatase inhibitors cocktail (0.2 mM PMSF; 10 μg/ml aprotinin; 10 μg/ml leupeptin; 1 mM Na_3_VO_4_; 1 mM NaF) (Sigma Aldrich, St. Louis, MO) and kept at -80°C until WB analysis performed as previously described [21]. Briefly, after blocking the membranes were incubated with primary antibodies (COX-2, EGFR, PDGFR, VEGFR, p-ERK1/2, cyclin D1, p65, p27, and actin). Details about dilution for each antibody are listed in Table 1. Membranes were incubated with horseradish peroxidase-conjugated secondary antibodies (1:3,000 dilution) and immunoreactive bands were visualized with an enhanced chemiluminescence system (Pierce Biotechnology, Rockford, IL).

### Animal study

All animal studies were performed in accordance with approved protocols by the UT IACUC committee as previously described in details [21]. The primary K9TCC cells were subcutaneously implanted in athymic nude mice (n = 5/cell line, 1.5×10^6^ cells/mouse with 1:1 Matrigel/PBS) to confirm tumorigenic behavior of tested TCC cells. Human UMUC-3 cells were used as positive controls (3×10^6^ cells/mouse with 1:1 Matrigel/PBS). The tumors lengths were measured by a digital caliper once per week for 3 weeks. After 3 weeks, the xenograft K9TCC tumors were dissected from mice, fixed, and evaluated by IHC.

## Results

### Characterization of primary K9TCC tumors

Primary K9TCC cell lines were established from four female dogs with confirmed diagnosis of urinary tract TCC. The canine patients with bladder cancers already had advance stages of TCC at the time of diagnosis.

**Figure 1:**
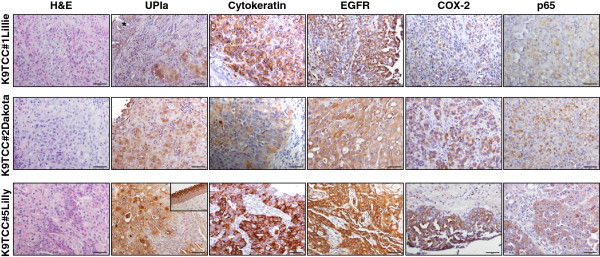
**Characterization of K9TCC tumors *in vivo*.** The histology of K9TCC tissues were confirmed by H&E staining. The expressions of cytokeratin, EGFR, COX-2 [21], p65, and UPIa in K9TCC#1Lillie, K9TCC#2Dakota, and K9TCC#5Lilly (brown color) detected by IHC. Objective 20× with scale bar 50 μm. (*) shows normal urethral urothelium in K9TCC#1Lillie, and inset image shows normal bladder urothelium of K9TCC#5Lilly tissue sample.

Primary K9TCC#1Lillie cell line was established from biopsy sample obtained from urethra of a 16-year-old female Pointer dog. The representative histology of the K9TCC#1Lillie by H&E staining is shown in Figure 1. The UPIa, a marker for urothelial cells [14], was expressed in normal urethral urothelial cells (asterisk in Figure 1) with moderate expression of UPIa detected in neoplastic K9TCC#1Lillie cells as shown in Figure 1. We confirmed the urothelial-cell origin of isolated K9TCC#1Lillie by positive expression of cytokeratin, E-cadherin [21], and UPIa using IHC. We confirmed the expression of COX-2 [21], EGFR, and p65 in this primary tumor by IHC (Figure 1).

Primary K9TCC#2 Dakota cell line was established from biopsy sample of urinary bladder of a 13-year-old female Bichon Fries dog. The representative histology of the K9TCC#2Dakota by H&E staining is shown in Figure 1. We confirmed the epithelial-cell origin by positive expressions of cytokeratin, UPIa, and E-cadherin in Figure 1. The K9TCC#2Dakota cells showed strong COX-2 [21], p65 (NF-κB), UPIa, and diffused EGFR expressions (Figure 1).

Primary K9TCC#4Molly cell line was established from biopsy sample of urinary bladder of a 10-year-old female Maltese dog. Unfortunately, the immunohistochemistry analysis of above mentioned markers were not performed in K9TCC#4Molly due to an insufficient size of biopsy sample obtained during the cystoscopy.Primary K9TCC#5Lilly cell line was established from biopsy sample of urinary bladder of a 13-year-old female mixed-breed dog. The representative histology of the primary tumor of K9TCC#5Lilly by H&E staining is shown in Figure 1. Cells showed the strongest expressions of COX-2, EGFR, and p65 as compared to other tested K9TCC (Figure 1). The strong expression of UPIa was detected in normal bladder urothelial cells, especially in normal terminally-differentiated superficial urothelial cells (inset in Figure 1 in K9TCC#5Lilly for UPIa). The decreased intensity of UPIa expression was detected in neoplastic K9TCC#5Lilly confirming the cell-origin from bladder urothelium.

### Doubling time and morphology of tested primary K9TCC cells

**Figure 2:**
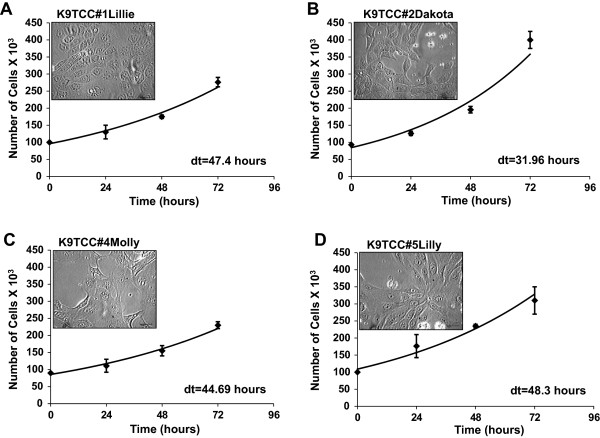
**Doubling time and morphology of primary K9TCC cells.** The doubling times for **(A)** K9TCC#1Lillie (passage #6) was dt = 47.4 hours, **(B)** K9TCC#2Dakota (passage #5) was dt = 31.96 hours, **(C)** K9TCC#4Molly (passage #4) was dt = 44.69 hours, and **(D)** K9TCC#5Lilly (passage #4) was dt = 48.3 hours. Values were represented as the mean ± S.E. (n = 3 for each time point). The representative images of K9TCC cell morphology were taken by phase-contrast microscope and are shown in insets of graphs. Objective 20× with scale bar 50 μm.

Cell proliferation of established primary K9TCC cells was further characterized using doubling time. The doubling time of the K9TCC cell lines was calculated by counting trypsinized cells each 24 hours for 3 days. The doubling time (dt) for K9TCC#1Lillie was dt = 47.4 hours (Figure 2A), for K9TCC#2 Dakota was dt = 31.96 hours (Figure 2B), for K9TCC#4Molly was dt =44.69 hours (Figure 2C), and for K9TCC#5Lilly was dt = 48.3 hours (Figure 2D).Morphology of tested K9TCC cells was evaluated by phase-contrast microscope as shown in insets of Figure 2. All tested K9TCC cells had polygonal morphology, except K9TCC#4Molly cells that showed more flatten appearance of cells. K9TCC cells were variable in sizes containing single or multiple nucleoli and cytoplasmic vacuoles (K9TCC#4Molly).

### The expression profile of cancer-related markers in four primary K9TCC cells

**Figure 3:**
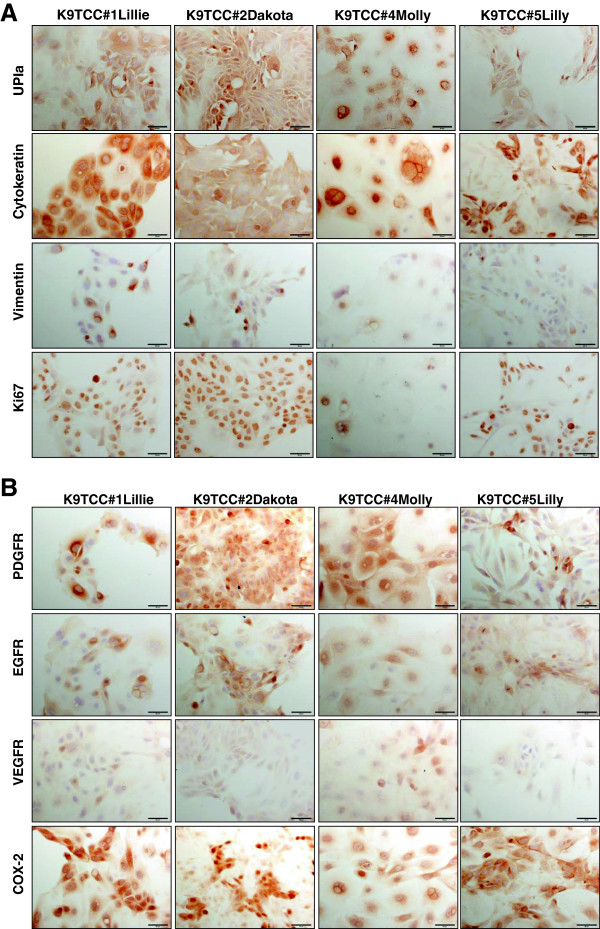
**Characterization of primary K9TCC cells *in vitro*.** The expression of UPIa was used to confirm the urothelium cell-origin of established K9TCC cells K9TCC#1Lillie, K9TCC#2Dakota, K9TCC#4Molly, and K9TCC#5Lilly (brown color) *in vitro* using ICC. Cytokeratin was expressed in the membrane in all tested K9TCC cells and confirmed the epithelial-cell origin of established K9TCC cells. Weak expression of vimentin was observed only in K9TCC#1Lillie and K9TCC#2Dakota cells. Ki67 expression was positive in nucleus, confirming that K9TCC cells were undergoing cell-cycle division. All tested K9TCC cells showed strong COX-2 and PDGFR expressions. Moderate expressions of EGFR and low expressions of VEGFR were detected in all tested K9TCC cells. Objective 20× with scale bar 50 μm.

**Figure 4:**
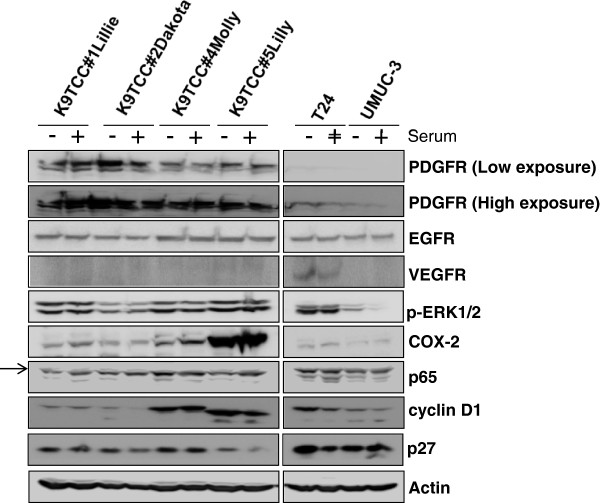
**The expression profile of cancer-related markers in primary K9TCC cells.** K9TCC cells were grown in presence and absence of serum for 24 hours and collected cell lysates were analyzed by WB. The expression levels of PDGFR, EGFR, VEGFR, p-ERK1/2, COX-2, p65, cyclin D1, and p27 were evaluated. Actin was used as loading control. The arrow shows the specific band for p65.

The expressions of several cancer-related markers were tested in the established primary K9TCC cells and compared to human T24 and UMUC-3 cells by ICC and WB analysis. The semi-quantitative analysis of the ICC data are shown in Table 2.All tested K9TCC cells expressed the urothelium-specific marker UPIa by ICC as shown in Figure 3. We tried to detect the expression of UPIa antibody using WB analysis; however no UPIa band was detected using this antibody (data not shown). All tested K9TCC cells showed strong expressions for cytokeratin by ICC, confirming the epithelial-cell origin, as shown in Figure 3. K9TCC had very weak expression of cytoplasmic vimentin by ICC, therefore confirming that these cells were epithelial and not mesenchymal cell-origin (Figure 3). Ki67 was used as a marker for cell proliferation and was strongly detected in three tested K9TCC, except K9TCC#4 Molly that had only moderate expression of Ki67 by ICC as shown in Figure 3.Tyrosine kinase receptors play an important role in cancer regulation. In our study, we tested the expression levels of the several most common tyrosine kinase receptors, such as PDGFR, EGFR, and VEGFR in K9TCC cells. Our ICC and WB data identified that PDGFR was more expressed in K9TCC#1Lillie and K9TCC#2Dakota than in the two other K9TCC#4Molly and K9TCC#5Lilly as shown in Figure 3 and Figure 4; respectively. EGFR was moderately expressed in all tested K9TCC and human TCCs by ICC (Figure 3) as well as by WB (Figure 4). VEGFR was not detected in tested K9TCC (Figure 3 and Figure 4) and only moderate expression of VEGFR was observed in human T24 cells by WB (Figure 4). The expression of active (phosphorylated) extracellular signal regulated kinases (p-ERK1/2) as one of the downstream activators of tyrosine kinase receptors were expressed in all tested TCC by WB as shown in Figure 4. Interestingly, the expressions of p-ERK1/2 were less in K9TCC#2Dakota and UMUC-3 than in the other tested TCC.

**Table 2:** Semi-quantitative analysis of cancer-related markers by ICC

	**K9TCC#1Lillie**	**K9TCC#2Dakota**	**K9TCC#4Molly**	**K9TCC#5Lilly**
UPIa	+	+++	+	++
Cytokeratin	+++	+++	++	++
Vimentin	-	-	-	-
Ki67	+++	+++	+	+++
PDGFR	++	+++	+++	++
EGFR	+	+	+	+
VEGFR	_	_	_	_
COX-2	+++	++	++	+++

Higher levels of COX-2 are associated with higher grade tumors [22]. COX-2 was highly expressed in all TCC in perinuclear locations by ICC as shown in Figure 3, with highest expression of COX-2 in K9TCC#5Lilly detected by WB (Figure 4). Human T24 cells were used as positive control and UMUC-3 as negative control for COX-2 expression (Figure 4). The expression of the p65 (NFκB), as one of the downstream target of COX-2 signaling pathway, was detected in all tested TCC as shown in Figure 4.Cell-cycle-related proteins, such as cyclins and their inhibitors were also evaluated in tested K9TCC. Cyclin D1 is well known as a cell-cycle regulator of G1 phase of the cell cycle. As shown in Figure 4, all tested TCC expressed cyclin D1 with highest expression in K9TCC#4Molly and K9TCC#5Lilly. Interestingly, the expression of p27, a cell-cycle dependent kinase inhibitor, was highly expressed in tested K9TCC except of K9TCC#5Lilly. Actin was used as a loading control for WB analysis (Figure 4).

### Tumorigenic behavior of primary K9TCC cells

**Figure 5:**
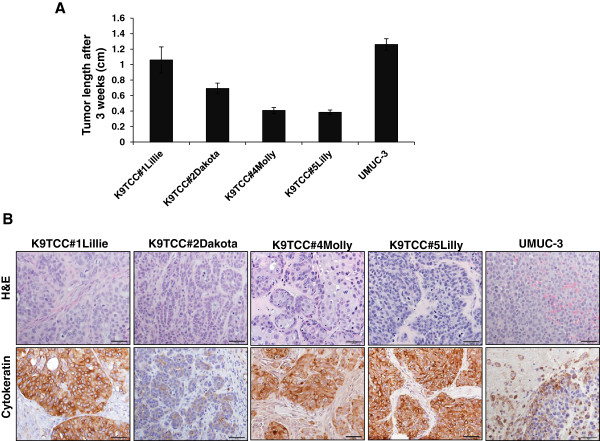
**Tumorigenic behavior of K9TCC cells. (A)** The primary K9TCC cell were subcutaneously inoculated in athymic mice (n = 5/cell line, 1.5 × 10^6^ cells/mouse with 1:1 Matrigel/PBS) to confirm tumorigenic behavior of tested K9TCC cells. Human UMUC-3 cells were used as positive controls (3 × 10^6^ cells/mouse with 1:1 Matrigel/PBS). Tumor length was measured after three weeks. Values were represented as the mean +/-S.E. of tumors length (n = 5 mice). **(B)** Representative images of H&E and cytokeratin expression (brown color) of K9TCCs and UMUC-3 xenograft tumors. Objectives 20× with scale bar 50 μm.

In our previously published study, we confirmed *in vivo* tumorigenic behavior of two K9TCC cell lines: K9TCC#1Lillie and K9TCC#2Dakota [21]. Human UMUC-3 cells were used as a positive control [21]. In this study, we confirmed tumorigenic behavior of two additional K9TCC cell lines: K9TCC#4Molly and K9TCC#5Lilly as shown in Figure 5. K9TCC#1Lillie xenograft tumors reached a size of approximately 1 cm in length within three weeks. K9TCC#2Dakota xenograft tumors reached a size of approximately 0.7 cm in length within three weeks. The smallest size of K9TCC#4Molly and K9TCC#5Lilly xenograft tumors (approximately 0.4 cm) were observed 3 weeks after inoculation in cells (in Figure 5A). The h-UMUC-3 xenograft tumors had the largest size of approximately 1.2 cm in length after three weeks as shown in Figure 5A. The histology of all tested K9TCC xenograft tumors confirmed that tumors were of epithelial-cell-origin and formed lobules, clusters, cysts with partially necrotic centers. K9TCC#4Molly xenograft tumors contained large cells as shown by H&E staining *in vivo* (Figure 5B) similarly as previously observed *in vitro* by ICC (Figures 2 and 3). Cytokeratin expressions were stronger in K9TCC#1Lillie, K9TCC#4Molly, and K9TCC#5Lilly xenograft tumors as compared to K9TCC#2Dakota and UMUC-3 xenograft tumors. High expression of E-cadherin and COX-2 in K9TCC#1Lillie and K9TCC#2Dakota xenograft tumors and no expression of COX-2 in UMUC-3 xenograft tumors were confirmed in our previously published study [21]. Histology of UMUC-3 xenograft tumor identified that UMUC-3 cells were not forming any pattern of clusters or lobules, (Figure 5B) suggesting that UMUC-3 cells are less differentiated and more aggressive carcinoma compared to the established K9TCC carcinomas. This observation was confirmed by counting the mitotic figures in tested TCC xenograft tumors. The mitotic figures in tested K9TCC xenograft tumors was lower, with range from 6 to 8 per high power fields (40×), as compared to h-UMUC-3 cells with approximately 16 mitotic figures/high power fields (40×).

## Discussion

Dogs with spontaneous tumors are still an underexploited tool to make rapid advances in human cancer prevention, diagnosis, and therapy. Dogs with naturally occurring cancers provide an important step for successful translation of novel imaging and therapeutic agents from rodents to human clinical applications (review *in Press*). The average age of the affected dogs with spontaneous cancers is 8.4 years, which corresponds to an average age of 50 years for humans. This suggests that as in humans, spontaneous carcinomas in dogs are influenced by age and environmental factors. Cancer heterogeneity and the ability to study the responses of naturally occurring cancer to therapy in a timely manner are further advantages of a canine model [23]. The value of this approach has been increasingly recognized in the studies identifying the cancer-associated markers, the environmental risk factors, understanding tumorigenesis, and the development of novel cancer therapeutics [24,25]. The major limitation of companion animal models of cancer is for studies evaluating the monoclonal antibodies for cancer therapy [26].

Despite advances in treatment of K9TCC, median survival times reported for prospective clinical trials have never exceeded 1 year regardless of the treatment modality [27]. Surgery and radiation therapy are useful treatment modalities in some selected cases of K9TCC [27]. Currently, combined protocols of chemotherapy with targeted therapies, such as the non-steroidal anti-inflammatory drugs (piroxicam), show promising inhibition of bladder TCC cells growth *in vitro*[9]. Piroxicam in combination with cisplatin or carboplatin induces remission in canine TCC more often than cisplatin [28] or carboplatin [29] alone.

Fluorocoxib A, a novel optical imaging agent that specifically detects COX-2-expressing cancers [30], was evaluated to detect canine bladder cancer using two primary K9TCC cell lines: K9TCC#1Lillie and K9TCC#2Dakota [21]. The results from our previous study showed that fluorocoxib A selectively binds to COX-2–expressing primary K9TCC cells *in vitro*, COX-2–expressing K9TCC xenografts tumors in nude mice *in vivo*, and heterogeneous K9TCC during cystoscopy *in vivo*[21]. Newly established primary canine and feline oral squamous cell carcinoma cell lines (K9OSCCAbby and FeOSCCSidney) were characterized and effects of novel tyrosine kinase inhibitor, Masitinib (AB Sciences) in combination with non-steroidal anti-inflammatory drugs were evaluated using this model *in vitro*[31]. Those are examples of the utilization of newly established primary canine cancer cell lines as model for human and canine cancers. Canine cancer models are valuable and efficient for evaluation and translation of novel imaging and therapeutic drugs to human medicine.

Despite the disadvantages, cancer cell lines have been, and will continue to be, the model system of the cancer *in vitro*. Very few K9TCC cell lines are currently available to perform such studies [11], therefore we established and characterized four new primary K9TCC cell lines to be used for *in vitro* and *in vivo* studies using athymic nude mice model. In our study, we evaluated cell cycle markers of established K9TCC cells as previously published by Knapp et al. [11], and in addition, we also evaluated the expression of UPIa and several growth factor receptor tyrosine kinases by ICC and WB analysis. We compared the expression of several cancer-related markers in primary tumor tissues obtained from the dogs *in vivo* with primary K9TCC *in vitro* by ICC and WB analysis to confirm that expression profiles of proteins were not altered during tissue culture preparation and expansion of cells. The tumorigenic behavior of tested K9TCC cell lines was confirmed by formation of xenograft tumors in the athymic mice. One of the limitation of primary cancer cell lines is that they might stop proliferating partially due to critical telomere shortening. In our experiments, we have utilized our primary K9TCC cell lines with passages# 10 to 20; however we didn’t not noticed any changes in behavior of those cells *in vitro* passing potential “crisis”.

The animal models (rodent and companion animal models) that recapitulate the nature of human cancers are major prerequisite for rapid bench-to-bedside translation of novel anti-cancer drugs and imaging agents that showed promised in cancer cells *in vitro* to human medicine (review In Press). The decision of which model of cancer to use depends on the stage of drug discovery. However, the final proof of concept for efficacy and safety of novel therapeutic and imaging drugs lies in humans.

## Conclusions

Spontaneously occurring cancers in pets share similar molecular and clinical characteristics with human cancers. Four new primary K9TCC cell lines (K9TCC#1Lillie, K9TCC#2Dakota, K9TCC#4Molly, and K9TCC#5Lilly) were characterized as epithelial-cell origin with confirmed cytokeratin, E-cadherin, and UPIa expressions. The positive expression of COX-2, PDGFR, and EGFR markers were also confirmed using ICC and WB analysis. The established primary K9TCC can be used to test novel targeted imaging and therapeutic agents for bladder cancers in dogs and people.

## Abbreviations

COX-2: Cyclooxegenase-2; EGFR: Epidermal growth factor receptor; ERK: Extracellular regulated kinase; H&E: Hematoxylin and eosin; ICC: Immunocytochemistry; IHC: Immunohistochemistry; K9TCC: Canine transitional cell carcinoma; PDGFR: Platelet-derived growth factor receptor; RTK: Receptor tyrosine kinases; TCC: Transitional cell carcinoma; UPIa: Uroplakin Ia; VEGFR: Vascular endothelial growth factor receptor; WB: Western blotting.

## Competing interests

The authors have no competing interest.

## Authors’ contributions

KR performed the laboratory experiments; performed WB, ICC, and cell proliferation assays; acquired images of cells in culture; assisted with *in vivo* mince experiments; performed the statistical analysis; and drafted the manuscript. MC conceived and designed the study; performed part of IHC staining; performed *in vivo* mice experiments; acquired digital images of IHC and ICC staining; and assisted with writing of manuscript. Both authors have read and approved the final version of the manuscript.

## Pre-publication history

The pre-publication history for this paper can be accessed here:

http://www.biomedcentral.com/1471-2407/14/465/prepub
